# Exploring the barriers of adherence to dietary recommendations among patients with type 2 diabetes: A qualitative study in Iran

**DOI:** 10.1002/nop2.558

**Published:** 2020-07-16

**Authors:** Firoozeh Mostafavi‐Darani, Fereshteh Zamani‐Alavijeh, Behzad Mahaki, Arash Salahshouri

**Affiliations:** ^1^ Department of Health Education and Promotion School of Health Isfahan University of Medical Sciences Isfahan Iran; ^2^ Department of Biostatistics School of Health Kermanshah University of Medical Sciences Kermanshah Iran; ^3^ Department of Health Education and Promotion School of Public Health Ahvaz Jundishapur University of Medical Sciences Ahvaz Iran

**Keywords:** barriers, diabetes mellitus, Iran, patients, qualitative research, type 2 diabetes; diet

## Abstract

**Aims:**

Type 2 diabetes is a major global health concern, and its prevalence is rapidly increasing throughout the world. The present study was conducted to explore the experiences of patients and healthcare providers to identify the social barriers to patients' adherence to their recommended diet and thus help the design of future interventions.

**Design:**

This study was conducted as a qualitative study with content analysis approach.

**Methods:**

The present qualitative study was conducted from November 2016–July 2017. Data were collected through 38 unstructured in‐depth interviews with 33 T2D patients and their treatment supervisors and field notes. The interview transcripts were coded using the MAXQDA 10 software. To extract categories and themes, the thematic analysis approach was used. We followed the COREQ Checklist to ensure rigour in our study.

**Results:**

The analysis of the study revealed the emergence of five categories of perceived barriers including social priorities and rivalries, family's food habits, poor social support, social impasses and dominant food patterns.

## 1 INTRODUCTION

Type II diabetes (T2D) is a major global health problem (Banerjee, Nema, Dhas, & Mishra, 2017), and its prevalence is rapidly increasing throughout the world (Hu et al., 2015). According to an International Diabetes Federation (IDF) report, 382 million adults in the world had diabetes in 2013 (Han, Kim, Kim, Lee, & Cho, 2017) and this figure is expected to reach 592 million by 2035 (Guariguata et al., 2014). The mentioned statistic also suggests that most of the diabetes patients will have T2D (Han et al., 2017). Currently, more than 4 million people have diabetes in Iran and this figure triples every 15 years (Esteghamati et al., 2014; Zendehtalab, Vaghei, & Emamimoghadam, 2013). According to the experts' unofficial predictions, the diabetes population of Iran will reach nine million by 2020 (Rohani et al., 2016). However, the prevalence of T2D is estimated to be 8.6% in Iran (Nosratabadi, Halvaiepour, Yousefi, & Karimi, 2016). The increasing prevalence of diabetes leads to extensive treatment costs. In this regard, in the United States, the total cost of DM amounts to $2108/patient per year (Leon & Maddox, 2015), because diabetes is associated with an increased risk of cardiac, renal and neurological diseases, visual impairment and early mortality (Baraz, Zarea, & Shahbazian, 2017; Noshad, Afarideh, Heidari, Mechanick, & Esteghamati, 2015).

## 2 BACKGROUND

Self‐management refers to the actions that people with chronic diseases can undertake to manage their symptoms, self‐treatment and compliance with behavioural changes recommended by healthcare professionals (Choi, Song, Chang, & Kim, 2014). Adherence to a healthy diet, performing physical activity and adherence to the prescribed medications are three basic principles of diabetes management (Reisi et al., 2017). The management of the disease is largely dependent on the active participation of the patients in adherence to the recommended diet (Halali, Mahdavi, Mobasseri, Jafarabadi, & Avval, 2016). According to literature, patients play the most important role in treatment and control of diabetes (Aris, Blake, & Adams, 2017). Therefore, the IDF has placed great emphasis on patients' self‐management for controlling blood sugar and reducing the risk of disease‐related complications (Ebadi Fard Azar, Hedari, & Solhi, 2016). Adherence to a healthy diet has been recommended as a major (Al Sayah, Majumdar, Williams, Robertson, & Johnson, 2013), first (Halali et al., 2016) and the most difficult (Chechlacz et al., 2009) step in diabetes management. Previous studies have revealed that patients with diabetes often refrain from proper self‐care (ALhariri, Daud, Almaiman, & Saghir, 2017; Reisi et al., 2017) and compliance with the advice given by the health professionals are low (Funnell, 2006; Reisi et al., 2017). The results of previous studies also show that many patients fail to adhere to their prescribed diets (Ganiyu, Mabuza, Malete, Govender, & Ogunbanjo, 2013; Grammatikopoulou et al., 2017; Parajuli, Saleh, Thapa, & Ali, 2014). Although the level of non‐compliance with T2D diet is still unclear, studies suggest that this level varies from 2.2%–87.5% (Ibrahim, Attia, Sallam, Fetohy, & El‐Sewi, 2010; Parajuli et al., 2014; Shokair, 2010). Adherence to a healthy diet can be affected by various intrapersonal, interpersonal and social factors (Mohebi et al., 2013) and can be improved in patients by identifying and removing these barriers.

Determinants of adherence to a healthy diet in patients with diabetes, especially from the perspective of patients and the healthcare providers (HCPs), can be of great assistance in the design of interventional programmes for promoting healthy eating. The present study seeks to explore the experiences of T2D patients and their treatment supervisors to identify the social barriers to patients' adherence to their recommended diet and help with the design of future interventions.

## 3 METHODS

### 3.1 Setting

The study settings were diabetes clinics, healthcare centres and local healthcare units in Isfahan, Iran.

### 3.2 Research design

The study was conducted as a qualitative study with content analysis approach, using in‐depth interviews with patients with T2D and HCPs to explain the barriers of adherence to dietary recommendations among patients with type 2 diabetes. An inductive approach was used, and data were analysed using thematic analysis (Hassani, Ezbarami, Tafreshi, & Majd, 2017).

### 3.3 Study participants

Purposeful sampling with the maximum variation (Tewahido & Berhane, 2017) was applied to select 33 T2D patients & HCPs. Therefore, a heterogeneous group of patients with T2D with a wide range of background characteristics (Gender, age, education status, etc.) was selected. The inclusion criteria were having a history of high or uncontrolled blood sugar, a history confirmed T2D for more than 3 months for patients and willingness to take part in the study. The participants were accessed from the patient registration books in diabetes clinics, healthcare centres and local healthcare units. To obtain more comprehensive and in‐depth data, other informers were also interviewed, including HCPs with at least 6‐month experience of diabetes care, educational backgrounds in nutrition and willingness to participate in the programme. HCPs included four disease prevention and control experts, two nutritionists, two community health workers and two general practitioners. After selecting patients from the registration books, treatment supervisors performed the necessary coordination for the interview. Next, the patients were informed about the goals of the study by a researcher nurse. After interviewing 29 participants, the answers were repetitive. Since all patients were illiterate, four literate patients were selected and interviewed to ensure the participants' maximum variation in terms of education. The unstructured in‐depth interviews were continued until data saturation, meaning that no new findings and code were found from participants' interviews. Therefore, after interviewing 33 people, it was assumed that no new data would be added to the results. A total of 38 people were invited to participate in the study. Two persons were excluded for not having proper physical and mental conditions to answer the interviewer's questions and one removed for sudden death. Also, two persons were reluctant to participate in the study and left it.

### 3.4 Data collection

The process of interviewing patients and collecting data was performed from November 2016–July 2017. Unstructured in‐depth interviews were used to collect data from participants. Field notes were also recorded immediately after each interview, as another way of data collection and to assess the participants' non‐verbal behaviours and interactions with others. The first (FM) and fourth authors (AS) were responsible for conducting the interviews. Before the interviews, demographic data were collected (Table 1). Eventually, 38 interviews with 33 people (including 23 patients and 10 HCPs) were performed face‐to‐face in the healthcare centres (21 interviews), the patients' homes (6 interviews) and at the diabetes clinic (8 interviews).

**Table 1:** The participants' demographic details

Variable	Diabetes patient	Health professionals	Total
	*N* (%)	*N* (%)	*N* (%)
Gender			
Male	10 (43.4)	8 (0.8)	18 (54.5)
Female	13 (56.5)	2 (0.2)	15 (45.4)
Age group			
20–40	4 (17.3)	9 (0.9)	13 (39.3)
41–60	11 (47.8)	1 (0.1)	12 (36.3)
60 and older	8 (34.7)	0 (0)	8 (24.2)
Education			
Illiterate	16 (69.5)	0 (0)	16 (48.4)
Junior high school	3 (13)	0 (0)	3 (9.0)
High school diploma	3 (13)	0 (0)	3 (9.0)
Associate degree	1 (4.3)	3 (0.3)	4 (12.1)
Bachelor's degree	0 (0)	4 (0.4)	4 (12.1)
Master's degree	0 (0)	1 (0.1)	1 (3)
PhD	0 (0)	2 (0.2)	2 (6)
T2DM history in 1st‐degree relatives			
Yes	18 (78.2)		
No	5 (21.7)		
Occupation status			
Employed	4 (17.3)		
Retired	4 (17.3)		
Housewife	10 (43.4)		
Unemployed	5 (21.7)		
Time since diagnosis			
<1 year	2 (8.6)		
1–5 years	5 (21.7)		
>5 years	16 (69.5)		
Household income			
Inadequate to cover living expenses	16 (69.5)		
Sufficient to cover living expenses	7 (30.4)		
Complications of diabetes			
Yes	8 (34.7)		
No	15 (65.2)		
Physical activity			
Regular	5 (21.7)		
Irregular	8 (34.7)		
No physical activity	10 (43.4)		
Drug use and smoking			
Yes	4 (17.3)		
No	19 (82.6)		
Visit by doctor			
Regular	14 (60.8)		
Irregular	9 (39.1)		
Medications			
Metformin only	8 (34.7)		
Metformin & glibenclamide	8 (34.7)		
Metformin & insulin	7 (30.4)		

The duration of each interview depending on the interviewee's interview conditions varied from 20–60 min. For example, the vagueness of the interviewees' responses and the need to provide additional explanations led to longer interview time. Secondly, it depends on the interest and willingness of the interviewee. For example, when the participant did not have enough time to answer the questions, the length of the interview was shorter and, if necessary, the interview continued again at another time.

The interviews were held without the attendance of the patients' family members. At the start of each interview, the participants were briefed on the study objectives and then were asked to give their written consent for having the interviews recorded. At the end of each interview, participants' consents were obtained a second time regarding the issues that arose during the interviews. Although the research team highly stressed the confidentiality of all the data, two of the patients declined to be interviewed. The interviews with patients began with introductory questions to create a friendly atmosphere and continued with phrases such as “please talk about your disease and conditions,” “what you mean by…,” “tell me more about,” etc. The next questions were unplanned and were posed based on the interviewer' experiences and discussion of the subject. The interviews were recorded using a voice recorder and were transcribed (on paper) and then typed up verbatim at the first opportunity. After typing the interviews and their review, the authors conducted a second interview with five of the cases in response to the need for further information.

### 3.5 Data analysis

The audio‐recorded interviews were first transcribed verbatim and then typed into Microsoft Word. The typed‐up interviews (154 pages) and field notes were imported to Qualitative data analysis software and Mac OS X (MAXQDA 10) and analysed using Hassani et al.'s (2017) thematic analysis model. Each interview text was scrutinized several times, and the first‐level codes (812 codes) were extracted by breaking down the texts. These initial codes were then categorized according to their similarities and differences and the second‐level codes (15 codes) were then produced by naming each category, repeating the categorization, combining the similar codes and adding new emerging codes. Finally, the emerged themes were categorized. In fact, the inductive method was employed in the present study. When all the data were encoded and categories were created, the authors also assessed each category in terms of saturation. The data from 38 interviews with 33 participants were ultimately analysed. Given their previous experiences with qualitative research, the first (FM) and fourth authors (AS) contributed more to the data analysis. According to these authors, data saturation occurred when they could no longer discern any new information and perception. All the stages of interviewing, typing the interviews and encoding were done in Persian, as the native language of the participants. Eventually, the categorized codes were translated into English. In addition, further discussions with the qualitative research experts enabled AS and FM to be reflexive of assumptions and biases that may have influenced the research process.

### 3.6 Scientific trustworthiness of the results

We followed the COnsolidated criteria for REporting Qualitative research (COREQ): 32‐item checklist to ensure rigour in our study (see Supplementary File). To evaluate and enhance the scientific trustworthiness or rigour of the results, the criteria recommended by Lincon and Guba were used; that is, credibility, transferability, dependability and confirmability (Dalvand, Dehghan, Rassafiani, & Hosseini, 2018). Therefore, to evaluate and enhance the credibility of the findings, the attempt was made to select the participants with the maximum diversity of experiences and the sampling continued until data saturation. In order to increase the content validity, member checking was used and then the transcribed and encoded data were returned to the participants in order to confirm and comment. Transferability of data was provided by offering a comprehensive description of the subject, participants, data gathering and data analysis. Also, to increase the dependability of the research results, an external observer examined the data carefully (external checking). To enhance the confirmability, several research collaborators were put into use.

### 3.7 Ethics approval and consent to participate

The ethics committee of the Isfahan University of Medical Sciences approved the present study (Project No: 396522 and code of Ethics: IR.MUI.REC.1396.3.522). The consent of the relevant authorities was obtained before beginning the study. At the beginning of the study, the participants were briefed on the study objectives, interview methods; confidentiality of the data and the voluntary participation and the right to end their involvement at any point from the study and also their written informed consent for the participation was obtained. The time and place of the interviews were arranged with the participants, so that they could have ample time to discuss the subject.

## 4 RESULTS

Most of the patients in the study were female (56.5). Also, 47.8% of patients were in the age group 41–60 years. Table 1 presents the demographic characteristics of the patients.

It was inferred from participants' statements that social factors are major barriers to the adherence of T2D patients to a healthy diet. These factors were found to have five categories, including social priorities and rivalries, family's food habits, poor social support, social impasses and dominant food patterns (Table 2).

**Table 2:** The codes, subcategories and categories of social barriers to the adherence of patients with T2D to a healthy diet

Code		Subcategory	Category
Continuing eating out of respect for the guests		Pleasing others and gaining their respect	Social priorities and rivalries
Accompanying the husband in eating to make him feel comfortable			
Preparing meals according to other people's palate			
Ignoring the diet out of respect for the host			
Prioritizing the children's economic problems over one's own diabetes diet		Prioritizing the problems of the family members	
Prioritizing the children's illness over one's own diabetes diet			
Prioritizing the children's nutritional needs over one's own diabetes diet			
A family habit of consuming fatty foods			Family's food habits
A habit of eating red meat in family gatherings			
The lack of financial support from the family for a healthy diet	Poor financial support	Poor family support	Poor social support
The lack of financial support from the family for visiting a nutritionist			
The non‐acceptance of diabetes diets by other family members	The family's poor emotional support and understanding of the patient		
Experts spending little time on training the patients	Poor information and counselling support	Poor support from the healthcare providers	
The lack of a close relationship with the patient	Poor emotional support		
The lack of time for preparing healthy foods due to work engagements		Unconducive work conditions	Social impasses
Fatigue and hunger due to work conditions			
Other people's sympathy		People's reactions	
Other people's insistence			
Family members getting upset about the patient preparing different foods			
Others regarding the disease hereditary in the family	Fearing the stigma of diabetes		
Others regarding the patient with diabetes as defective			
The popularity of eating red meat in the region		The popularity of unhealthy foods	Dominant food patterns
The popularity of consuming animal lard in the region			
The popularity of bread as a staple food in the region			
The popularity of rice and pasta as a staple food in the region			

### 4.1 Social priorities and rivalries

This category has two subcategories, including pleasing and gaining the respect of others and giving priority to the problems of the family members. Most patients revealed that they occasionally ignored their recommended diet and to please and gain the respect of others. Accompanying the guests in eating until they finished their food, accompanying the spouse in eating to make them comfortable, accounting for the guests' palate in food preparation and eating non‐diet food as a guest to gain the respect of the host were the main reasons presenting by the patients.

One of the participants said: “Sometimes, I overeat offering more respect to my guests. When we have guests, I have to keep them company in eating until they finish, so they won't feel shy. That is why I sometimes don't follow my diet” (A 59‐year‐old man with T2D).

Another participant said that her husband's comfort was the reason for her failure to follow her diet: “I add potatoes to the stew when my husband is around and keep him company in eating to make him feel comfortable” (A 35‐year‐old woman with T2D).

Some patients mentioned prioritizing their family problems over their disease as a reason for not adhering to their diabetes diet. The reasons given by them included the priority of the family's economic problems and the children's illness and their nutritional requirements.

Another participant considered his children's nutritional needs are more significant than his own disease: “For my diabetes, I put diet foods on the table and my children like to eat them too, but these foods will not be enough for their growth. So, for their sake, I have to eat non‐diet foods” (A 32‐year‐old man with T2D).

### 4.2 Family's food habits

The effect of the food habits persisting from former lifestyles in the family and the particular eating habits related to family events and outings is another social barrier to patients' adherence to a healthy diet. Participants' experiences suggest that some patients cannot observe a healthy diet because of the food habits developed in them over the years in their family. One of the participants said: “We've been using animal fat and red meat in the family since we were kids and so have gotten used to these two things and can't give them up” (A 74‐year‐old man with T2D).

One of the treatment supervisors said: “How can someone who has eaten red meat and fatty foods all his life and never had fish be expected not to eat red meat anymore and have fish instead? These patients are dependent on these foods” (A 35‐year‐old male disease expert).

A woman with T2D said: “We have these weekly family get‐togethers in which we barbeque red meat.”

One of the nutritionists about the role of family's food habits on patients said: “People in this area do not have a reasonable dietary pattern. They have learned to just feed their belly rather than adhere to a balanced food intake. Therefore, this culture of food left over from childhood also affects adherence of patients to dietary recommendations during disease.”

### 4.3 Poor social support

Another important social barrier to the adherence of patients with T2D to a healthy diet is poor social support. Some patients considered poor financial support from the family and their lack of understanding and emotional support for the patient and their disease among the most significant barriers to their adherence to a healthy diabetes diet.

One of the patients argued: “How can I buy fruits and vegetables when my husband cannot bring home sufficient money?” (A 44‐year‐old woman with T2D).

About the family's lack of understanding for the patient and his disease, one of the nutritionists asserted: “Given their poor understanding of the patient and his illness, some families don't accept diabetes diets. So, the patients are forced to cook their meals according to the family members' palate and eat that food themselves too” (A 24‐year‐old male nutritionist).

Some participants considered poor emotional, information and counselling support on the part of the healthcare team as a barrier to the patients' adherence to a healthy diet. One of the patients said: “Doctors don't spend enough time providing us with information about a good diet. So, we are naturally unable to discern all unhealthy foods from healthy options” (A 54‐year‐old man with T2D).

One of the experts discussed the health care providers' failure to establish a good relationship with the patients and its role in the patients' adherence to their diet: “At first, I was unable to establish that good relationship with my patients and so couldn't understand why the patients didn't comply with the diet I'd given them. With some experience, I learned that I can get better results by spending more time with my patients and connecting with them” (A 24‐year‐old male nutritionist).

### 4.4 Social impasses

Social impasses are social conditions, problems or barriers that impede the patients' adherence to a healthy diet and make the patients with diabetes feel unable to perform self‐care for their disease. This category consists of two subcategories:

#### 4.4.1 Unconducive work conditions

In unconducive work conditions, which are associated with a general lack of time, fatigue and hunger caused by excessive workloads, the patients become unable to manage their disease and adhere to their diet. One of the experts said: “Due to work issues and some associated problems, one of my patients was unable to observe his diet; because he was too busy to spend any time preparing healthy foods for him or to eat on time” (A 26‐year‐old male nutritionist).

Two of the patients stated: “Because of work, I had my breakfast very late every day and by then I felt so weak and hungry that no amount of food could satiate my hunger” (A 44‐year‐old woman with T2D).

“I am very busy at work and get home so late that hunger does not let me prepare a healthy food. So, I have to eat whatever I am given, whether healthy or not” (A 32‐year‐old man with T2D).

#### 4.4.2 People's reactions

Another factor discussed by the participants as a barrier to diet adherence was the lack of support and knowledge related to diabetes among relatives and the patients´ local community, which not only prevented them from adequately supporting healthy eating behaviours of patients, but sometimes even led to inappropriate reactions to such behaviours. Some members of the family were saddened because of the need to provide a diet that is different from the food of the rest of the family for patients and since they worked harder to do that, they sometimes expressed dissatisfaction with this issue.

A 66‐year‐old woman with T2D explained: “I eat the same food that I cook for the family, because my child or husband may get upset that I eat a different food.”

Also, some people due to their lack of familiarity with the type of disease and how to care for it and only for the pity and sympathy, invited the patients to eat unhealthy foods by an immoderate level of insistence. Therefore, these were among the reactions that often made the patients ignore their diet when eating in groups and occasionally in the family. One of the participants said: “I was invited to someone's house a few days ago and the host begged me to have a few mouthfuls, so I had a few spoons of rice. But here nonstop insistence and sympathy made me ignore my diet” (A 32‐year‐old man with T2D).

Another patient described: “Sometimes when I'm invited to a party and don't eat the foods on the table because of my condition, they insist so much that I am forced to have a little bit of their food” (A man with T2D).

Fear of the stigma of diabetes discerned in people's reactions is among the most important social barriers to the patients' adherence to a healthy diet. Fearing that people might regard their disease hereditary in their family and treat them as defected because of it, the patients conceal their disease from others as much as possible and some even avoid eating diet foods in the presence of others in order to keep their illness concealed. Moreover, they occasionally do not allow their treatment supervisors to follow up on their health. One of the patients asserted: “I did not follow my diet in the presence of others so that they wouldn't find out because people think that this disease is hereditary and all the members of my family have it” (A 54‐year‐old woman with T2D).

One of the treatment supervisors said: “One of my patients asked me not to go to his house or shop to take care of his illness because he did not want his neighbours to find out about his disease by seeing me there” (A 24‐year‐old male nutritionist).

Another patient said: “People regard a patient with diabetes as defective, so I do not follow my diet when I am with others and eat whatever they eat, so they won't find out about my disease” (A 38‐year‐old woman with T2D).

### 4.5 Dominant food patterns

The dominant food pattern of a region has a major role in shaping the nutrition type of the patients. If these patterns are unhealthy, they cause the patients not to adhere to their diabetes diet and make them neglect their disease control. According to the participants, the popularity and consumption of certain unhealthy foods in the region affected by the dominant food patterns impede their adherence to a healthy diet.

One of the patients revealed: “I still use animal fat because I'm a villager and all villagers have always used these foods. We are all animal farmers in the village; so, eating large amounts of red meat is common. It is the staple food of the people in this region and all of us mostly eat this food” (A 74‐year‐old man with T2D).

One of the treatment supervisors said: “The food culture of the people of this region largely affects a patient's adherence to a healthy diet. For instance, bread is a staple for the people in this region and is consumed with most foods in large amounts. This also applies to patients with diabetes and affects their adherence to a healthy diet” (A 24‐year‐old nutritionist).

## 5 DISCUSSION

The present qualitative study examined the experiences of patients with T2D and their treatment supervisors regarding the social barriers to patients' adherence to a healthy diet. In general, by analysing qualitative data, five categories of barriers, including social priorities and rivalries, family's food habits, poor social support, social impasses and dominant food patterns were identified.

In most interviews, pleasing others, gaining their respect and giving priority to the family's economic problems, the children's illness and their nutritional needs over own illness were frequently raised by the participants as subcategories the social priorities and rivalries against adherence to a healthy diet.

Since the culinary culture of Iran emphasizes particular gestures of respect towards others, self‐care recommendations that do not match the patients' culture cannot positively affect their self‐care process. This issue is so dominant in the present study that the most patients stated that they sometimes have the same foods as others, especially in parties and celebrations, out of respect for the host and the other guests and they accompany others in eating non‐diet foods to avoid upsetting the host and to please them. Accompanying own guests until they are full, so that they will not feel deserted or shy to eat is a common custom in the Iranian culture that was discussed by several of the participants as a social barrier. Occasionally, efforts to please the guests and gain their respect made the patients ignore their healthy diet by giving priority to the guests' palate and neglecting their own nutritional requirements during food preparation.

The patients prioritized resolving their family's economic problems by taking measures to have their children recover from illness, attending to their children's nutritional needs by observing their own diet and controlling their own disease. In such a situation, they seem to attach greater importance to their social problems than their diet and disease (Basu & Garg, 2017). Therefore, if the limitations of resources and social problems are not improved as an underlying factor, it can have a long‐term impact on adherence to the healthy diet and increasing the risk of complication related to diabetes. Accordingly, until the patients maintain these priorities on their mind, any advice and encouragement about self‐care that fails to take account of these issues will prove useless. Although the results do not directly refer to the social role of patients with diabetes as one of the factors of non‐adherence to a healthy diet, it can be stated that social role is one of the factors to be considered, indirectly. An explanation for this result is that having a parent role alone has many responsibilities, including the provision of family members' needs, which can act as a barrier against self‐care behaviours in married patients. In contrast to the present study, the results of a study on patients with T2D indicated that that the dietary control in married patients was higher than that of unmarried patients (saeidinejat, 2014). However, the results of a cross‐sectional study on patients with type 2 diabetes who were 89.8% married showed that most of them had a mean dietary practice score moderate or good (Shamsi, Shehab, AlNahash, AlMuhanadi, & Al‐Nasir, 2013). Therefore, prioritizing the family members' needs can make patients reluctant to change their long‐term behavioural patterns.

Food habits are rooted in the cultural, environmental, social and religious elements of every society (Sapkota, Jo‐anne, Gwynn, Flood, & Aslani, 2017). Some of the participants believed that the food habits and behaviours learned in the family affect their adherence to diet in adulthood even when they are ill. A common cultural component of Iranian family living is holding picnics where red meat is barbequed and served in a picnic. Many patients with diabetes are still bound to the food habits acquired through their family and eat non‐diet foods in their family gatherings as a non‐dying custom. This is a significant issue because the identification of these harmful habits enables their elimination or replacement of them with better nutritional habits. The important point here is families' inclination towards undesirable food habits, such as eating barbecued red meat and strategies should be adopted to replace this habit with healthier ones. Of course, this food habit is common in the Middle East, as in Iran and in the study of Nachvak et al. (2018), it is referred to as one of the most popular foods in the Middle East. Therefore, educating patients about disease and diet can provide a basis to changing food habits. Also, considering the family's effective role in following the dietary habits of patients, it seems that attracting the participation of families of patients in the field of healthy nutrition as an enabling factor can facilitate this goal. However, since it is probably not possible to stop barbecued red meat consumption for patients in the region, replacing this type of food with barbecued lean white meat, which is somewhat similar in terms of taste and manner of preparation, can help to healthy eating and control of the disease.

Moreover, due to the nature of T2D and its treatment, patients' success in the management of their disease and adherence to their diet requires support from the family and the society. Social support, in general and family support, in particular, is the vital part of successful diabetes control (Taher et al., 2016). In this regard, many of the participants considered poor social support as one of the social barriers to adherence to a healthy diet. The mentioned finding concurs with the results obtained in a study by Maryam Mataji Amirrood, Taghdisi, Shidfar, and Mahmood Reza (2014), which found support from the family and people around to be one of the best strategies for promoting healthy food behaviours in the society. In the present study, the patients recalled the lack of financial, emotional and information/counselling support from the family, friends and treatment supervisors as a barrier to a healthy diet. Shao, Liang, Shi, Wan, and Yu (2017) found that the more support the patients receive from their spouses and relatives, the more they become devoted to self‐care. A study by Pieroth, Radler, Guenther, Brewster, and Marcus (2017) also demonstrated a positive relationship between social support and the overall quality of diet among middle‐aged and older men in the United States.

In another work, Di Matteo and Miller illustrated that people with diabetes require the support from other people. Family members have a major role in the patients' adherence to their treatment and are considered a source of support without whom the patient's adherence to treatment will be difficult and sometimes impossible (Miller & DiMatteo, 2013). In line with these findings, the patients in the present study also stressed the supportive role of the family members and the medical team with regard to training and resolving their information requirements and clarifying their ambiguities in relation to a healthy diabetes diet. Therefore, in order to achieve better therapeutic outcomes, it seems that the family should be involved as an essential source of support for the self‐care process, as well as educating and justifying patients in a family way, so that the patient's family's financial and emotional supports can be created through changing the family's attitude. On the other hand, with the use of specialized and trained diabetes nurses, who have more time to respond to the patient, enough time to answer the patient's questions can be considered.

According to the present findings, a good relationship between the health care providers and the patients that fosters trust in the medical team and their instructions can lead to a greater adherence to the nutritional recommendations. A study by Ivanov revealed that successful diabetes care requires teamwork among the doctors and the patients. One part of this teamwork is a successful doctor–patient relationship that aims to improve patient satisfaction and adherence to the treatment (Beverly, Worley, Prokopakis, & Ivanov, 2016). Although self‐care is a significant factor in the management of chronic disease, however, due to the fact that nutritional behaviours and an adherence of a healthy diet are affected by the ecological patterns, it is required to address the environmental support which can be achieved through health system, health team (physician, nurse, etc.), work settings, social organizations, institutions and etcetera. Hence, it is necessary to pay special attention to promote dietary adherence in patients with diabetes (Jardim et al., 2018).

In the current study, some of the treatment supervisors discussed their positive experiences of building a proper relationship with the patients and its favourable effect on their self‐care efforts. Similarly, other studies indicated that a successful diabetes care requires a successful teamwork between the doctors and the patients; moreover, the doctor–patient relationship and their joint decision‐making are a part of this teamwork whose significance in improving patient satisfaction, treatment adherence and health outcomes was established. The mentioned teamwork facilitates the communication of medical information between the doctor and the patient (Beverly et al., 2016). Previous studies demonstrated that positive social relationships between patients and the health service team can improve health behaviours such as choosing healthier foods (Pieroth et al., 2017).

Therefore, since nurses spend more time in caring for patients than other treatment teams, they have a clinically unique role in patient care. Therefore, if the treatment team and especially the nurses have a good relationship with patients, they can take on their core duties, including evaluating patients' concerns, understanding, empathy, patient support and informing about illness and treatment. Therefore, it is imperative that nurses' education managers and authorities pay attention to the promotion of communication skills of nurses.

Another important barrier to the patients' adherence to a healthy diet was social impasses, which included unconducive work conditions and others' reactions, which might create difficult and stressful conditions for the patient and impede their adherence to the diabetes diet. The participants noted others' poor reactions and negative attitude towards diabetes as a factor that made them ignore their diet in collective settings and gatherings. Such treatment and society's dominant attitude can have negative effects on the patients' adherence to their diet and also affect their performance at their job, their income and any other aspect of their life.

In line with the present results, Chew, Shariff‐Ghazali, and Fernandez (2014) noted the lifelong need of patients with diabetes for psychological support since the patients' psychological health has a key role in their self‐care behaviours. The mentioned support can be provided by the healthcare system, the family and the society. Healthcare providers and the media can contribute to transform the society's negative attitude and thus help the patients adhere to self‐care behaviours more properly such as observing a healthy diet.

Dominant food patterns comprised one of the barriers extracted in the present study. A region's food pattern is the result of human cultures and environmental factors such as the availability of food items, the people's purchasing power, food preparation skills and food promotions in the media (Leng et al., 2017). In the present study, the patients argued that their reason for non‐adherence to a healthy diet was their inability to buy certain food items. Moreover, in rural areas, some local products are abundant (such as animal lard and red meat), and therefore, their inhabitants are more inclined to consume these products. In other words, the availability and lower cost of local produce are among the factors shaping the patients' food patterns in a given region. Food behaviours of some patients are affected by the dominant regional food culture. In this regard, some people may not observe their diabetes diet due to bread and rice being a staple food in the region and the habitual consumption of these items in all menus. Similar to the current study, other studies also confirmed the association between diet habits and dietary patterns of patients with T2D and its management (Jannasch, Kröger, & Schulze, 2017; Shamsi et al., 2013). Another study also considered the preference for local and traditional foods as barriers to the adherence of patients with T2D (Ebrahim, De Villiers, & Ahmed, 2014). Therefore, emphasizing the patient's food patterns and their underlying factors may result in predicting some strategies for the removal of the existent barriers to the patient's adhesion of a healthy diet.

In general, the present findings on the social barriers of the adherence of patients with T2D to a healthy diet can be used by HCPs and the society in designing comprehensive programmes for empowering patients in adhering to a healthy diet. Also, since the recommended healthy nutrition to the general population is similar to the diet recommended for those with diabetes especially in the early stages of the disease, the findings are of importance to HCPs for planning health promotion programmes for unhealthy eating at the community level.

### 5.1 Limitations of the Study

The funding and timing limitation restricted us to conduct a comprehensive review study. Therefore, the results of the current study are not truly representative of the entire population. The non‐cooperation of some of the treatment supervisors with the researchers and reluctance of some of the patients to participate were among the study limitations; however, they were partly overcome by briefing the participants on the study objectives and ensuring them of the confidentiality of their data.

## 6 CONCLUSION

The present study identified new dimensions of social barriers affecting the nutrition of patients with T2D, such as social priorities, rivalries and social impasses, which are scarcely mentioned in previous studies. Therefore, when planning a therapeutic approach for these patients, these factors should be considered by the treatment team members, including nutritionists, nurses and physicians. On the other hand, considering that the results of the study identified lack of family support as an important barrier to healthy diet, it is suggested that treatment teams and especially diabetes nurses, engage the family of patients in the process of self‐care as an essential source of support (emotional, financial and informational) for achieving better therapeutic outcomes.

Disseminating the findings can increase the awareness of nurses and the treatment team of patients with diabetes' nutrition issues. The results of the study demonstrated that the dominant food patterns play an important role in patient adherence to their diet. Since the food patterns of patients are rooted in the cultural, environmental, social and religious factors of each society, the healthcare providers are advised to consider the nutritional requirements of patients with diabetes in their nursing intervention programmes. In addition, our findings suggest that poor social support is one of the major barriers to diabetes self‐care. Thus, the deployment of effective strategies to provide emotional, informational and financial support to the children, spouses and other family members of patients in nursing intervention programmes can be an effective way in improving the nutrition of patients.

The findings of the current study also highlight the issues that can be considered as barriers to adherence to a healthy diet in different countries. Although the barriers or facilitators on this subject vary in each community and require examination in any social and cultural context, it is crucial to address interventions programmes in the diabetes control and outline the general principles in the instructions and guidelines for global diabetes control.

Therefore, the findings highlight the need to have a multidimensional approach for controlling the disease in general and promote adherence to a healthy diet in people with diabetes in particular.

## CONFLICT OF INTEREST

The authors declare that they have no competing interests.

## AUTHOR CONTRIBUTIONS

BM and AS recruited participants. AS and FM made substantial contributions to the conception and design of the study. AS and FZ analysed and interpreted interview data. AS and FZ drafted the manuscript. AS and FM were the supervisor of the study and provided the final article.

## Supporting information

Supplementary File
