# Supplementary material for: Exploring the barriers of adherence to dietary recommendations among patients with type 2 diabetes: A qualitative study in Iran
Source: Nurs Open. 2020 Jul 16;7(6):1735–45. doi: 10.1002/nop2.558 (PMC7544840; doi:10.1002/nop2.558)
Supplement: Supplementary file 1 — Supplementary File [file NOP2-7-1735-s001.pdf]

Consolidated criteria for reporting qualitative studies (COREQ): 32-item checklist

YOU MUST PROVIDE A RESPONSE FOR ALL ITEMS. ENTER N/A IF NOT APPLICABLE

**Topic**	**Item No.**	**Guide questions/description**	**Reported on page No**
**Domain 1: Research team and reflexivity**			
Personal Characteristics			
Inter viewer/facilitator	1	Which author/s conducted the inter view or focus group?	40 ,page 4
2. Credentials	**2**	What were the researcher's credentials? E.g. PhD, MD	In title page
3. Occupation	**3**	What was their occupation at the time of the study?	In title page
4. Gender	**4**	Was the researcher male or female?	In title page
5. Experience and training	**5**	What experience or training did the researcher have?	33, page 5
Relationship with participants			
6. Relationship established	**6**	Was a relationship established prior to study commencement?	N/A
7. Participant knowledge of the interviewer	**7**	What did the participants know about the researcher? e.g. personal goals, reasons for doing the research	3 ,page 4
8. Interviewer characteristics	**8**	What characteristics were reported about the inter viewer/facilitator? e.g. Bias, assumptions, reasons and interests in the research topic	39-41, page 5
**Domain 2: study design**			
Theoretical framework			
9. Methodological orientation and Theory	**9**	What methodological orientation was stated to underpin the study? e.g. grounded theory, discourse analysis, ethnography, phenomenology, content analysis	41-42, page 3
Participant selection			
10. Sampling	**10**	How were participants selected? e.g. purposive, convenience, consecutive, snowball	46-47, page 3
11. Method of approach	**11**	How were participants approached? e.g. face-to-face, telephone, mail, email	22-23, page 4
12. Sample size	**12**	How many participants were in the study?	22, page 4
13. Non-participation	**13**	How many people refused to	14, page 4

		participate or dropped out? Reasons?	
Setting			
14. Setting of data collection	**14**	Where was the data collected? e.g. home, clinic, workplace	23-24, page 4
15. Presence of non-participants	**15**	Was anyone else present besides the participants and researchers?	43, page 4
16. Description of sample	**16**	What are the important characteristics of the sample? e.g. demographic data, date	48, page 3
Data collection			
17. Interview guide	**17**	Were questions, prompts, guides provided by the authors? Was it pilot tested?	49, page 4
18. Repeat interviews	**18**	Were repeat inter views carried out? If yes, how many?	3-4, page 5,five of the cases
19. Audio/visual recording	**19**	Did the research use audio or visual recording to collect the data?	44-45, page 4
20. Field notes	**20**	Were field notes made during and/or after the inter view or focus group?	33-36, page 4
21. Duration	**21**	What was the duration of the inter views or focus group?	29, page 4
22. Data saturation	**22**	Was data saturation discussed?	8-9, page 4
23. Transcripts returned	**23**	Were transcripts returned to participants for comment and/or correction?	51-53, page 6
**Domain 3: analysis and findings**			
Data analysis			
24. Number of data coders	**24**	How many data coders coded the data?	25, page 5
25. Description of the coding tree	**25**	Did authors provide a description of the coding tree?	29, page 5
26. Derivation of themes	**26**	Were themes identified in advance or derived from the data?	29-30, page 5
27. Software	**27**	What software, if applicable, was used to manage the data?	22,page 5
28. Participant checking	**28**	Did participants provide feedback on the findings?	N/A
Reporting			
29. Quotations presented	**29**	Were participant quotations presented to illustrate the themes/findings? Was each quotation identified? e.g. participant number	Yes, Throughout the results section
30. Data and findings consistent	**30**	Was there consistency between the data presented and the findings?	Yes
31. Clarity of major themes	**31**	Were major themes clearly presented in the findings?	Yes, Throughout the results section
32. Clarity of minor themes	**32**	Is there a description of diverse cases or discussion of minor themes?	Yes, throughout the section of and "Discussion"
